# A risk factor for early wheezing in infants: rapid weight gain

**DOI:** 10.1186/s12887-019-1720-3

**Published:** 2019-10-15

**Authors:** Lijuan Yin, Ye Song, Yongfang Liu, Zehui Ye

**Affiliations:** 1 Department of Respiratory Center, Children’s Hospital of Chongqing Medical University, No.136 Zhongshan Second Road, Yuzhong District Chongqing, 400014 People’s Republic of China; 2 Department of Pediatrics, The First Affiliated Hospital of Air Force Military Medical University, Xi’an, 710000 People’s Republic of China; 3 Department of Nutrition, Children’s Hospital of Chongqing Medical University, Chongqing, 400014 People’s Republic of China

**Keywords:** Infant, Wheezing, Rapid weight gain, Risk factor

## Abstract

**Background:**

The aim of this study was to investigate the correlation between rapid weight gain and early wheezing.

**Methods:**

This study screened 701 infants with lower respiratory tract infection who were no more than 4 months from Jan 1st to Dec 31st in 2018. According to weight-for-age Z-value (WAZ), these infants were divided into the considerably slow weight gain group (group I), the normal weight gain group (group II) and the excessively rapid weight gain group (group III), respectively. The clinical characteristics, weight growth speeds and serum lipid levels were analyzed, and multivariable Logistic model was conducted to select significant variables.

**Results:**

Our results showed that male (OR = 1.841, 95%CI: 1.233–2.751), family wheezing (OR = 5.118, 95%CI: 2.118–12.365), age (OR = 1.273, 95%CI: 1.155–1.403), eczema (OR = 2.769, 95%CI: 1.793–4.275), respiratory syncytial virus (RSV) infection (OR = 1.790, 95%CI: 1.230–2.604), birth weight (OR = 1.746, 95%CI: 1.110–2.746) and total cholesterol (TC) (OR = 1.027, 95%CI: 1.019–1.036) and ΔWAZ (OR = 1.182, 95%CI: 1.022–1.368) were associated with early wheezing. Results indicated that serum TC (*P* = 0.018) and ΔWAZ (*P* = 0.023) were positive correlation with wheezing days.

**Conclusion:**

Besides male, family wheezing, age, eczema, RSV infection, birth weight and TC, the rapid weight growth as a risk factor should be concerned in the early wheezing infants.

## Background

Wheezing, as a common clinical symptom of pediatric respiratory disease, is characterized by a continuous and sonorous voice in the expiratory phase, and sometimes appears in the inspiratory phase which results in increased respiratory rate [1]. Approximately 50% of children suffered from wheezing in infancy and childhood [2], and roughly 26% of 6265 babies had wheezing at least once within 18 months after birth from a prospective study [3]. Furthermore, about 30–40% of patients with recurrent wheezing were eventually diagnosed as bronchial asthma [4]. Therefore, it is necessary to explore the potentially risk factors to improve the pediatric respiratory system disease.

Infancy, especially the first year after birth, is one of peak periods of growth and development of the whole life. The rapid growth of the body signifies the further maturation and functional perfection of various organs. The pathogenesis of wheezing may be special and complicated in this period. Studies have confirmed that obesity and bronchial asthma suffered in adulthood, are closely connected with this period [5]. The previous study has manifested that weight-gain acceleration in early infancy was related to the increased risk of asthma symptoms in preschool children [6]. However, wheezing in infants is a heterogeneous disease, and the current mechanism of non-allergic wheezing is still unclear [7]. Several studies have reported that weight gain in infants is a risk factor for wheezing in childhood and even puberty [8–14]. To our best knowledge, however, there are few researches focusing on the relationship between the weight gain speed and wheezing in infants, especially early infantile.

Herein, we investigated the demographic and related clinical characteristics, weight growth speeds and serum lipid levels of infants with early wheezing after birth. The primary purpose of this study was, thus, to identify the rapid weight growth as a potential risk factor for the early wheezing.

## Methods

### Patients

Totally 701 infants admitted to Children’s Hospital of Chongqing Medical University were retrospectively screened in this study from Jan 1st to Dec 31st in 2018. This study was approved by the medical ethics committee of Children’s Hospital of Chongqing Medical University, and the approval number was No.73/2019.

Hospitalized infants with lower respiratory tract infection who were no more than 4 months were included from Jan 1st to Dec 31st in 2018.

Patients who accorded with the following criteria were excluded: (1) premature delivery (gestational age ≤ 37 weeks); (2) congenital cardiovascular anomaly; (3) bronchopulmonary dysplasia; (4) congenital tracheomalacia; (5) congenital stenosis of bronchus; (6) incomplete clinical data 1, such as lack of blood biochemical indexes, or blood samples with requirements unmatched.

### Data collection

The baseline data were collected including gender, age, birth weight and length, weight (ACS-20-YE electronic baby scale) and length (HX-II infant horizontal length measuring instrument) at admission, gestational age, delivery mode, feeding style, lactation diet, living environment, family history of obesity, allergy and wheezing, as well as history of individual eczema. The birth weight and weight at admission were analyzed to calculate the weight-for-age (WAZ) Z-value using WHO Anthro (version 3.0).

The data of illness condition were collected, such as the length of stay (LOS), date and season of onset, wheezing days, cases of severe wheezing, premier wheezing and repeated wheezing, days of intravenous hormone and common complications.

### Laboratory examination

The pathogenic indicators were tested via the bacterial culture of nasopharyngeal secretions in combination with drug sensitivity test, enzyme-linked immune-sorbent assay (ELISA) and polymerase chain reaction (PCR). The results consisted of respiratory syncytial virus (RSV), adenovirus (Adv), influenza virus A (IVA), influenza virus B (IVB), parainfluenza virus (PIV-1, 2 and 3), mycoplasma pneumoniae (MP), chlamydia pneumoniae (CT) and cytomegalovirus (CMV).

The serum biochemical indices were detected utilizing Backman AU5800 automatic biochemistry analyzer, including total cholesterol (TC), triglyceride (TG), low density lipoprotein (LDL) and high density lipoprotein (HDL). Only when the children with fluid diet were fasted at least 3–4 h could the levels of blood lipids be collected. The fasting time was 6 h at least if a child ate solid food as supplements. When hemolysis or lipemia occurred in the specimens, the blood would be re-extracted or directly excluded.

### ΔWAZ

The present study referred to the reported literature by Ong et al. [15], the equation is ΔWAZ = WAZ_at admission_ - WAZ_at birth_. The details are as follows: (1) ΔWAZ≥0.67 for excessively rapid weight gain; (2) -0.67 < ΔWAZ< 0.67 for normal weight gain; (3) ΔWAZ≤-0.67 for considerably slow weight gain. In accordance with the above standards, these infants were divided into the considerably slow weight gain group (group I), the normal weight gain group (group II) and the excessively rapid weight gain group (group III), respectively.

### Statistical analysis

Statistical analysis was performed using SPSS 18.0 (SPSS, Inc., Chicago, IL). Measuring data were presented as the mean ± standard and analyzed by ANOVA. Counting data were presented as n (%) with Chi-square or Fisher test. The relationship between wheezing and related indicators was investigated using multivariate Logistic regression analysis. The association between wheezing days and serum TC, TG, HDL, LDL, ΔWAZ was analyzed by multiple stepwise regression analysis. The equation for inclusion and exclusion criteria was 0.15. *P* < 0.05 was considered statistically significant.

## Results

### The baseline data in different weight growth patterns

A total of 735 cases were collected in this study, including 701 infants meeting the inclusion criteria. The primary characteristics of the participants were presented in Table 1. The parameters including age, gender, normal weight, low weight, overweight, eczema, severe wheezing, repeated wheezing, the length of onset (LOO), HDL and birth-weight Z score were no significantly statistical differences among the three groups (*P* > 0.05). The statistical differences among the three groups were shown in birth weight and length, weight and length at admission, family obesity, family wheezing, individual history of hypersensitivity, family hypersensitivity, wheezing, premier wheezing, the length of wheezing (LOW), LOS, TC, TG and LDL (*P* < 0.05).

**Table 1:** The baseline data of infants in different weight growth patterns

Variables	Group I	Group II	Group III	F/*χ*^*2*^	*P*
Case (n)	237	243	221		
Age (month, mean ± SD)	3.26 ± 2.52	3.23 ± 1.64	3.54 ± 2.24	1.385	0.251
Gender (n, %)					
Male	163 (68.78)	170 (69.96)	153 (69.23)	0.080	0.961
Female	74 (31.22)	73 (30.04)	68 (30.77)		
Birth weight (kg, mean ± SD)	3.38 ± 0.43	3.26 ± 0.44^#^	3.1 ± 0.40^*$^	25.708	< 0.001
Weight at admission (kg, mean ± SD)	5.35 ± 1.52	6.33 ± 1.28^#^	7.24 ± 1.71^*$^	87.504	< 0.001
Birth length (cm, mean ± SD)	50.11 ± 1.36	49.85 ± 1.51^#^	49.59 ± 1.36^$^	3.156	0.043
Length at admission (cm, mean ± SD)	58.1 ± 5.89	59.65 ± 5.80^#^	59.16 ± 6.08	4.224	0.015
Normal weight (n, %)	226 (95.36)	232 (95.47)	214 (96.83)	0.769	0.681
Low weight (n, %)	11 (4.64)	9 (3.70)	3 (1.36)	4.096	0.129
Overweight (n, %)	0 (0.00)	2 (0.82)	4 (1.81)	–	0.087
Obesity (n, %)	0	0	0		
Family obesity (n, %)					
Yes	49 (20.68)	28^#^ (11.52)	22^$^ (9.95)	12.910	0.002
No	188 (79.32)	215 (88.48)	199 (90.05)		
Family wheezing (n, %)					
Yes	14 (6.01)	17 (7.00)	28^*$^ (12.67)	7.534	0.023
No	219 (93.99)	226 (93.00)	193 (87.33)		
Individual history of allergy (n, %)					
Yes	55 (23.21)	53 (21.81)	90^*$^ (40.72)	24.913	< 0.001
No	182 (76.79)	190 (78.19)	131 (59.28)		
Eczema (n, %)					
Yes	60 (25.32)	70 (28.81)	64 (28.96)	0.996	0.608
No	177 (74.68)	173 (71.19)	157 (71.04)		
Family allergy (n, %)					
Yes	11 (4.64)	14 (5.76)	53^*$^ (23.98)	54.086	< 0.001
No	226 (95.36)	229 (94.24)	168 (76.02)		
Wheezing (n, %)					
Yes	105 (31.16)	128 (52.67)	136^$^ (61.54)	55.715	< 0.001
No	232 (68.84)	115 (47.33)	85 (38.46)		
Severe wheezing (n, %)					
Yes	28 (11.81)	34 (13.99)	37 (17.54)	3.011	0.222
No	209 (88.19)	209 (86.01)	174 (82.46)		
Premier wheezing (n, %)					
Yes	95 (40.08)	111 (45.68)	116^$^ (95.87)	110.451	< 0.001
No	142 (59.92)	132 (54.32)	5 (4.13)		
Repeated wheezing (n, %)					
Yes	4 (1.69)	8 (3.29)	12 (5.43)	4.863	0.088
No	233 (98.31)	235 (96.71)	209 (94.57)		
LOO (days, mean ± SD)	15.86 ± 16.69	14.73 ± 17.14	15.63 ± 18.74	0.276	0.759
LOW (days, mean ± SD)	7.44 ± 10.79	7.26 ± 9.92	11.02 ± 13.35^*$^	7.901	< 0.001
LOS (days, mean ± SD)	7.55 ± 3.79	6.65 ± 2.78^#^	6.78 ± 2.52^$^	5.868	0.003
TC (mg/dL, mean ± SD)	120.91 ± 32.46	127.81 ± 34.85^#^	140.24 ± 38.05^*$^	17.69	< 0.001
TG (mg/dL, mean ± SD)	56.49 ± 29.22	50.41 ± 21.96^#^	56.3 ± 27.45^*^	4.116	0.017
HDL (mg/dL, mean ± SD)	45.84 ± 19.97	47.47 ± 18.99	47.02 ± 19.47	0.445	0.640
LDL (mg/dL, mean ± SD)	59.03 ± 23.96	59.43 ± 21.84^#^	67.57 ± 25.80^*$^	9.254	< 0.001
Birth-weight Z score	−1.50 ± 1.21	−0.18 ± 1.04	0.88 ± 0.99	1.172	0.069

*LOO* The length of onset, *LOW* The length of wheezing, *LOS* The length of stay, *TC* Total cholesterol, *TG* Triglyceride, *HDL* High density lipoprotein, *LDL* Low density lipoprotein

-: Using Fisher test

^#^: Group I vs Group II, *P* < 0.05; ^*^: Group II vs Group III, *P* < 0.05; ^$^: Group I vs Group III, *P* < 0.05

We also investigated the seasons, systemic venous hormone injection and virus infections among wheezing patients in this study. The results suggested that the incidence of wheezing in spring (52.9%) was higher than other seasons, and the statistical differences were observed among the three groups (χ^2^ = 8.71, *P* = 0.013). The average days of systemic venous hormone injection were 2.07 ± 3.59, and there were no evident differences among the three groups (F = 2.922, *P* = 0.054). Totally 334 cases suffered from positive viral infections. The viruses with a highly positive infection rate were RSV (χ^2^ = 3.36, *P* = 0.187), PIV-3 (χ^2^ = 0.76, *P* = 0.68) and IVA (χ^2^ = 1.06, *P* = 0.59) in turn. The detectable rates of three viruses in infants with wheezing were shown no differences among the three groups (*P* > 0.05).

In addition, the characteristics of mothers in different weight growth patterns were analyzed in Table 2. The gestational age and pregnant frequency were statistically significant among the three groups (*P* < 0.05). However, no significant differences in parity, natural delivery, caesarean, exclusive breastfeeding, artificial feeding, mixed feeding, breastfeeding and complementary feeding were revealed among the three groups (*P* > 0.05).

**Table 2:** The characteristics of mothers in different weight growth patterns

Variables	Group I	Group II	Group III	F/*χ*^*2*^	*P*
Case (n)	237	243	221		
Gestational age (weeks, mean ± SD)	38.43 ± 1.48	38.25 ± 1.24	38.11 ± 1.27^$^	3.165	0.043
Pregnant frequency (n, mean ± SD)	1.87 ± 1.11	2.06 ± 1.43	2.24 ± 1.47^$^	4.450	0.012
Parity (n, mean ± SD)	1.38 ± 0.56	1.35 ± 0.56	1.42 ± 0.59	1.016	0.363
Natural delivery (n, %)	114 (48.10)	113 (46.50)	92 (41.63)	2.081	0.353
Caesarean (n, %)	123 (51.90)	130 (53.50)	129 (58.37)		
Exclusive breastfeeding (n, %)					
Yes	63 (26.58)	69 (28.40)	52 (23.53)	1.436	0.488
No	174 (73.42)	174 (71.60)	169 (76.47)		
Artificial feeding (n, %)					
Yes	68 (28.94)	77 (31.69)	59 (26.70)	1.405	0.495
No	167 (71.06)	166 (68.31)	162 (73.30)		
Mixed feeding (n, %)					
Yes	106 (44.73)	97 (39.92)	110 (49.77)	4.550	0.103
No	131 (55.27)	146 (60.08)	111 (50.23)		
Breastfeeding (n, %)					
Yes	169 (71.31)	166 (68.31)	162 (73.30)	1.426	0.490
No	68 (28.69)	77 (31.69)	59 (26.70)		
Complementary feeding (n, %)					
Yes	38 (16.03)	33 (13.58)	30 (13.57)	0.767	0.681
No	199 (83.97)	210 (86.42)	191 (86.43)		

^#^: Group I vs Group II, *P* < 0.05; ^*^: Group II vs Group III, *P* < 0.05; ^$^: Group I vs Group III, *P* < 0.05

### Multivariate logistic regression analysis for infants with early wheezing

The value of risk factors to predict early wheezing was further analyzed using multivariable Logistic regression, as presented in Table 3. Statistically obvious differences were shown in the male (OR = 1.841, 95%CI: 1.233–2.751), family wheezing (OR = 5.118, 95%CI: 2.118–12.365), age (OR = 1.273, 95%CI: 1.155–1.403), ΔWAZ (OR = 1.182, 95%CI: 1.022–1.368), eczema (OR = 2.769, 95%CI: 1.793–4.275), RSV infection (OR = 1.790, 95%CI: 1.230–2.604), birth weight (OR = 1.746, 95%CI: 1.110–2.746) and TC (OR = 1.027, 95%CI: 1.019–1.036). While no evidence demonstrated that countryside, TG, HDL, LDL may server as the risk factors for early wheezing infants in our research.

**Table 3:** Multivariate logistic regression analysis for infants with early wheezing

variables	*β*	*S.E*	*χ* ^*2*^	*P*	*OR*	95%CI
Male	0.610	0.205	8.886	0.003	1.841	1.233–2.751
Family wheezing	1.633	0.450	13.158	< 0.001	5.118	2.118–12.365
Countryside	−0.721	0.198	13.297	< 0.001	0.486	0.330–0.717
Age	0.241	0.050	23.723	< 0.001	1.273	1.155–1.403
ΔWAZ	0.167	0.074	5.064	0.024	1.182	1.022–1.368
Eczema	1.018	0.222	21.098	< 0.001	2.769	1.793–4.275
RSV infection	0.582	0.191	9.242	0.002	1.790	1.230–2.604
Birth weight	0.557	0.231	5.820	0.016	1.746	1.110–2.746
TC	0.027	0.004	38.333	< 0.001	1.027	1.019–1.036
TG	−0.006	0.004	2.159	0.142	0.994	0.987–1.002
HDL	−0.024	0.005	18.620	< 0.001	0.977	0.966–0.987
LDL	0.005	0.006	0.817	0.366	1.005	0.994–1.017
Constant	−4.230	0.928	20.803	< 0.001		

*RSV* Respiratory syncytial virus, *TC* Total cholesterol, *TG* Triglyceride, *LDL* Low density lipoprotein, *HDL* High density lipoprotein

### Regression analysis between wheezing days and serum lipid parameters

Regression analysis was used to assess the relationships between wheezing days and serum lipid parameters in Table 4. Results indicated that serum TC (*P* = 0.018) and ΔWAZ (*P* = 0.023) were positively correlated with wheezing days. Furthermore, no distinct connection was discovered between TG, HDL, LDL and wheezing days (*P* = 0.706, 0.056 and 0.500, respectively).

**Table 4:** Regression analysis between wheezing days and related indicators

Dependent variable	Independent variables	Regression coefficient (*β*)		*P*
		*β* value	Standard *β*	
Wheezing days^a^	TC	0.038	0.135	0.018
	TG	0.005	0.014	0.706
	HDL	−0.039	−0.075	0.056
	LDL	0.015	0.035	0.500
	ΔWAZ	1.193	0.126	0.023

*TC* Total cholesterol, *TG* Triglyceride, *LDL* Low density lipoprotein, *HDL* High density lipoprotein

^a^ Adjusting age, gender, birth weight, weight at admission, feeding style and lactation diet

## Discussion

Wheezing is a common chronic respiratory disease among children worldwide, and is also a worried event for parents and pediatricians because it may develop into asthma. The current study evaluated the potential predicting factors for infants with early wheezing. We retrospectively investigated 701 under 4 months infants hospitalized for lower respiratory tract infection. The findings suggested that the rapid weight gain could be a potential risk factor for infants with early wheezing.

Infancy, as a key stage, not only has profound effects on the physical growth and development, but also influences the occurrence and development of chronic diseases such as cardiovascular disease, hypertension and diabetes at various ages [5, 16, 17]. It is the fact that the first 6 months after birth is a high-risk period of overweight formation [18, 19]. Our study discovered the children in group III had lower birth weight and length, while those in group I had higher birth weight and length. When the infants were approximately at 4 months, the weight in group III was higher than other two groups, which had obvious catch-up growth trends. Theoretically, rapid weight gain may lead to different degrees of adipose tissue acquisition, and we observed the TC, LDL and TG in group III were distinctly higher than other two groups, in which TC was positively correlated with wheezing and wheezing days (*P* < 0.05), suggesting that lipid metabolism is enhanced with the increase of weight gain speed. Abnormal or disordered lipid metabolism is a common issue in adults and infants. However, to our best knowledge, there are lack of uniform standards for blood lipid detection in infants and normal standard value for the infants under 2 years old in China nowadays. In this study, no more than 20 cases of high blood lipids were found in all blood lipid indicators. The lipid disorder is temporary in infancy, and the level of lipid metabolism gradually decreases to the normal after one and a half years [20].

We discovered that the breastfeeding was a common feeding pattern, and no statistical differences were found among the three groups. Clinical observations displayed that the frequency of daily intake in group III was more than that other two groups. Nevertheless, it still needs further observation whether there exist differences in the growth speed of infants owing to lack of feeding knowledge. With the extension of breastfeeding time, the weight of mothers was on the decline, it may be self-fat catabolism of mothers leading to high breast milk fat content, which causes children to receive a high-fat diet from mothers. Imperfect synthetic function of HDL in small intestine and liver can result in enhancive cholesterol accumulation, elevated blood lipids containing the TC level, relatively increased LDL-carried cholesterol for the synthesis of cell membranes and steroid hormones, and accumulation of cell membranes and cholesterol of vital organs such as airway. Studies have reported that lipid metabolism has an impact on lung diseases. The data for animal experiments have observed that cholesterol transporters such as ABCG1, apoE and LXR are involved in immune defense of the respiratory tract, resulting in Th1/Th2 imbalance and elevated airway neutrophils and IL-17, which would lead to the susceptibility of respiratory tract infections [21, 22]. The clinical observation of this study discovered that neutrophils were mainly increased in bronchoalveoar lavage fluid (BALF) among wheezing infants, but not eosinophils. It is indicated that cholesterol transport promotes the neutrophil chemotaxis and upregulation of inflammatory cytokines which may participate in the development of wheezing [21].

Studies found that nearly half of children had wheezing within 1 year after birth, especially no more than 6 months [4]. To date scholars mostly had focused on investigating the effects of weight gain on wheezing or recurrent wheezing in different periods [12, 23–26], meanwhile several researchers found that growth speed was a risk factor for respiratory disease among children [27]. Our results displayed the there was no statistical significance among the infants with normal weight, low weight and overweight. It is indicated that the occurrence of wheezing is not relevant to weight [27], but may be associated with growth speed, which is in accordance with previous reports [10, 28, 29]. Evidences demonstrated that the weight growth speed might serve as a potential influencing factor in obesity [19], pulmonary function decline [29], bronchial asthma [30], cardiovascular disease [16], and type 1 diabetes [17] during infancy. Although various assessment criteria for wheezing have been used in recent years, rapid weight gain may also be considered as a hazardous element in wheezing [30, 31]. The data from Table 4 we obtained that wheezing days were positively correlated with the weight gain (*P* < 0.05). However, the mechanism between rapid weight gain and wheezing among infants is currently unclear.

Differences between intrauterine environment and postnatal environment may lead to the development of postnatal diseases [32, 33], and the compensatory growth appeared due to the intrauterine growth in unfavorable intrauterine environment. While rapid compensatory growth may be detrimental to the future health [13], rapid weight gain can result in relatively poor lung development, and airway stenosis can increase the incidence and severity of wheezing [13, 29]. Studies showed that rapid weight gain, especially in the first year after birth, was significantly related to the frequency and degree of wheezing [26, 34]. Early reports demonstrated that allergy and eczema can enhance the risk of wheezing or asthma [35, 36]. The data of our research revealed the proportions of the individual history of hypersensitivity and eczema were higher in group III. Furthermore, the viral infection as a suspicious factor was considered in several researches. RSV is the most common pathogen for infantile wheezing, with 46.5% of infection rate, but no statistical differences were discovered among the three groups. It is indicated that RSV infection was involved in the occurrence and development of wheezing, while it may be not a major factor in infants with early wheezing. In addition, excessively rapid weight gain may cause changes in lung development, including alveolar number, lung weight and immune system, especially adverse changes in immune function can increase the occurrence of asthma in children. In this study, we found an obvious family characteristic among infants in group III. The infants had a positive family history of wheezing, particularly first-degree relatives, suggesting family genetic predisposition in these individuals. The previous protocol mentioned that wheezing may be associated with the mitochondrial gene variation, especially the father’s genes [17]. These still need further in-depth researches and discussions in the future.

The superiority of this study was that few previous researches had investigated the relationship between the weight gain speed and wheezing in infants, especially early infantile wheezing in Chinese population. It was the fact that rapid weight gain may be a risk factor in infants with early wheezing, which is beneficial for pediatricians to effectively identify wheezing children. There were some limitations that should be warranted caution for interpreting the data in this work. Firstly, our investigation was a retrospective study based on a single center, which is lack of parental serum lipid, antenatal and postnatal smoking, and intrauterine growth retardation (IUGR) or small for gestational age infant (SGA) collection. Secondly, there were no clear biologic targets as observation indicators to indicate the association between weight growth speed and infants with early wheezing. Thus, multicenter studies with larger samples should be needed for further verification of the role of weight growth speed in early wheezing infants in clinic.

## Conclusion

The current study accessed the potential risk factors for early wheezing infants aged no more than 4 months. We analyzed the demographic and related clinical characteristics, weight growth speeds and serum lipid levels of infants with early wheezing after birth. Besides male, family wheezing, age, eczema, RSV infection, birth weight and TC, the rapid weight growth as a risk factor should be concerned in the early wheezing infants.

## Data Availability

The datasets used and/or analyzed during the current study are available from the corresponding author on reasonable request.

## Abbreviations

CI: Confidence interval
HDL: High density lipoprotein
LDL: Low density lipoprotein
LOO: The length of onset
LOS: The length of stay
LOW: The length of wheezing
OR: Odds ratio
RSV: Respiratory syncytial virus
TC: Total cholesterol
TG: Triglyceride
